# Application of biomedical informatics to chronic pediatric diseases: a systematic review

**DOI:** 10.1186/1472-6947-9-22

**Published:** 2009-05-05

**Authors:** Fatemeh Moeinedin, Rahim Moineddin, Alejandro R Jadad, Jemila S Hamid, Teresa To, Joseph Beyene

**Affiliations:** 1 Child Health Evaluative Sciences, Hospital for Sick Children, Toronto, Ontario, Canada; 2 Department of Community and Family Medicine, University of Toronto, Toronto, Ontario, Canada; 3 Dalla Lana School of Public Health, University of Toronto, Toronto, Ontario, Canada; 4 Department of Health Policy Management and Evaluation, University of Toronto, Toronto, Ontario, Canada; 5 Centre for Global eHealth Innovation, University of Toronto and University Health Network, Toronto, Ontario, Canada

## Abstract

**Background:**

Chronic diseases affect millions of children worldwide leading to substantial disease burden to the children and their families as well as escalating health care costs. The increasing trend in the prevalence of complex pediatric chronic diseases requires innovative and optimal delivery of care. Biomedical informatics applications play an important role in improving health outcomes while being cost-effective. However, their utility in pediatric chronic diseases has not been studied in a comprehensive and systematic way. The objective of this study was to conduct a systematic review of the effects of biomedical informatics applications in pediatric chronic diseases.

**Methods:**

A comprehensive literature search was conducted using MEDLINE, the Cochrane Library and EMBASE databases from inception of each database to September 2008. We included studies of any methodological type and any language that applied biomedical informatics to chronic conditions in children and adolescents 18 years of age or younger. Two independent reviewers carried out study selection and data extraction. Quality assessment was performed using a study design evaluation instrument to appraise the strength of the studies and their methodological adequacy. Because of heterogeneity in the conditions and outcomes we studied, a formal meta-analysis was not performed.

**Results:**

Based on our search strategy, 655 titles and abstracts were reviewed. From this set we identified 27 relevant articles that met our inclusion criteria. The results from these studies indicated that biomedical informatics applications have favourable clinical and patient outcomes including, but not limited to, reduced number of emergency room visits, improved knowledge on disease management, and enhanced satisfaction. Seventy percent of reviewed papers were published after year 2000, 89% of users were patients and 11% were either providers or caregivers. The majority (96%) of the selected studies reported improved outcomes.

**Conclusion:**

Published studies suggested positive impacts of informatics predominantly in pediatric asthma. As electronic tools become more widely adopted, there will be opportunities to improve patient care in a wide range of chronic illnesses through informatics solutions.

## Background

Chronic diseases, defined by the US Centers for Disease Control and Prevention (CDCP) as "illnesses that are prolonged, do not resolve spontaneously and are rarely cured completely", are responsible for two thirds of deaths worldwide [1]. It is estimated that more than 100 million people in North America live with one or more chronic illnesses, consuming more than 75% of the total health care resources [2]. The number of people with chronic illness is on the rise. It is estimated that by 2020, about 157 million people in North America (50% of the population), including children and adolescents, will be living with at least one chronic condition [3].

Chronic conditions in children have long-term effect on society, as they progress into adults with increasing demands for health care and disability programs, and decreasing participation in the work force as well as poor quality of life [4]. These chronic conditions cause stress on children and their families. Moreover, substantial amounts of time, energy and personal resources are required to cope with the physical and emotional burden [5]. National Health Interview Surveys on Disability from 1994–1995 indicated that 15% to 18% of children and adolescents in the USA were affected by some type of chronic conditions [4].

The rapid development and penetration of information and communication technologies (ICTs) has created opportunities for innovative ways to reduce the societal burden created by chronic diseases in children [6]. e-health, also known as health or biomedical informatics, is an evolving field, which strives for the acquisition, maintenance, retrieval and application of knowledge and information in health care science, services, research, and education to improve patient care [7].

To the best of our knowledge, there are no published systematic reviews on the role of biomedical informatics in the management of pediatric chronic diseases.

Previous systematic reviews of adult chronic disease have examined applications of home tele-monitoring [8], and information systems [2]. Pare et al [8] specifically studied tele-monitoring and Dorr et al [2] combined chronic conditions that are common in children. We included a wider range of interventions and reported effects on chronic diseases such as asthma and ADHD that are more common in children. In addition, Sanders et al [9] investigated one specific chronic disease, asthma. However, there appears to have been no systematic reviews providing a comprehensive review of a broad range of applications for both physical and mental chronic illnesses in pediatric settings. This study sought to fill this gap.

## Methods

### Search Strategy

We searched Medline (1950–September 2008), EMBASE (1982–September 2008), and the Cochrane database of systematic reviews (2008) for relevant studies using terms related to computer technology applications in pediatric chronic disease management. Each search required the presence of selected chronic diseases such as asthma, diabetes or autism in combination with any of the following terms: "medical informatics", "informatics", "decision support", or "computer assisted instruction" (detailed search strategy is available upon request).

### Study selection

We focused on studies evaluating applications of any web or computer-based information and communication technology designed to aid the clinical care of chronic illnesses in pediatric settings. We targeted studies that described or appraised a computer-based intervention or application to support clinical care of physical or mental chronic conditions. These included applications or systems used to diagnose or detect symptoms; applications that prevent or monitor symptoms; decision support systems, alert and reminder systems; and patient-centered education applications. Studies were included if (i) the participants were 18 years age or younger; (ii) there was a chronic condition present; (iii) there was at least one computer-based intervention; and (iv) there was a comparison between at least two groups. There was no restriction by language or publication status for inclusion in this systematic review.

Two reviewers (FM and RM) independently reviewed the titles and abstracts of publications identified by the search strategy and assessed each paper as either potentially relevant or not relevant based on study type, study design, subjects, setting, and intervention. The full text publication of a citation thought to be potentially relevant by either reviewer was retrieved. The reviewers independently verified the eligibility of the chosen articles. Disagreement between the two reviewers was resolved by consensus and in some cases by discussing with a third reviewer (JB).

### Data Extraction and Quality Assessment

Data were extracted using a structured data collection form we designed (this is available upon request). Data extracted included: author(s); date of publication; details of the intervention and control; details of participants; outcome measures reported in the primary studies; study duration; and summary measures such as mean, standard deviation and number of participants for the main outcome variable. In studies reporting more than one outcome measure, data were extracted for the primary outcome. Data were initially extracted by one reviewer (FM) and verified by the second reviewer (RM). Reporting adequacy was evaluated using an instrument that uses five criteria to identify potential causes of biases [9,10]: i) *Formation of study groups*-allocation of subjects between the control and intervention groups, ii) *Unit of allocation and analysis*, iii) *Baseline differences between study groups*, iv) *Type of outcome measure*, and v) *Completeness of follow-up*. A score of 0 to 2 was assigned to each criterion. Then, the scores were summed to give an overall assessment ranging from 0 representing the greatest potential for bias to 10 representing the least [9].

We further examined the characteristics of the studies by clinical setting (inpatient, outpatient, emergency department, patient home, multi-clinical settings or no settings), primary users (patients, healthcare professionals, parents, educators, or administrators), the target patient population, and the type of primary outcome variable. Outcome measures were considered either clinical (rates of emergency visits and hospitalization, dosage of drugs, symptom reduction and patient's quality of life) or non-clinical (patient education, knowledge and behavior, and adherence to protocols) [9].

## Results

### Description of Studies

**Figure 1:**
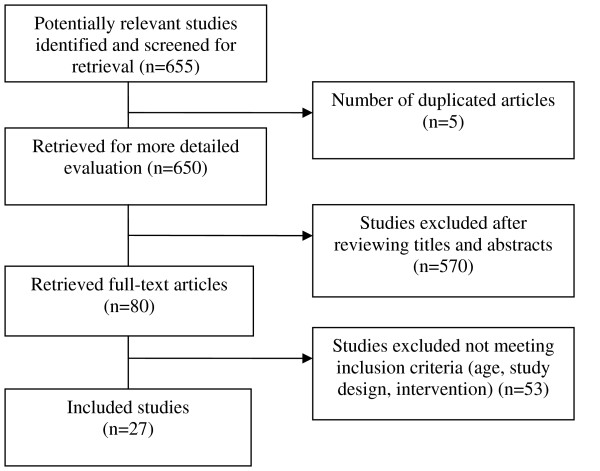
**Flowchart describing article selection process**.

MEDLINE and EMBASE search yielded 655 potentially relevant articles. From this set, a total of 27 publications met our inclusion criteria and were selected for review. Figure 1 provides a summary of the study selection process. Most of these studies (70%) were published after year 2000 and among studies that reported participants' gender, only one study did not include female participants. The age of participants ranged from one to eighteen years and almost all studies were quantitative with randomized, matched case-control, case crossover study designs. The mean and range of methodological adequacy scores for studies in the various chronic medical conditions were as follows: asthma 6.1 (4–8), cognitive disability 7.8 (6–9), autism 5 (4–7), pediatric oncology 6 (6-6), diabetes 6 (4–8), and other studies 7.5 (7–8). Selected publications evaluated the impact of applications on both clinical and non-clinical patient outcomes. Most studies (86%) took place in an outpatient environment. Eighty nine percent of users were patients and 11% were either providers or caregivers. Not all studies reported duration of intervention or follow up. Among those that reported them, the duration of the interventions ranged from 20 minutes to several weeks, while the duration of studies ranged from 4 days to 2 years. The majority (96%) of the selected studies reported improved outcomes. Additional file 1 displays the characteristics of the studies included in the systematic review.

### Asthma (8 Studies)

Chan et al [11] found improvement in quality of life, reduction in emergency department and hospital visits, infrequent rescue therapy and a high level of satisfaction with home telemonitoring. Shegog et al [12] showed significant improvement in knowledge and asthma self-management using a prospective pretest-posttest randomized intervention trial. Increase in knowledge of asthma self-management, reduction in emergency room visits and fewer hospitalizations were reported in relation to the computer game 'Watch, Discover, Think and Act' by Bartholomew et al [13]. This study showed that children using the program scored significantly higher on questions on steps of self-regulation, prevention and treatment strategies. Those children also demonstrated greater self-efficacy compared to children who did not use the program. Huss et al [14] conducted a randomized control trial in 7 to 12 year old children to evaluate the effects of a computer-assisted instructional game on asthma symptoms such as coughing, wheezing, shortness of breath, nighttime awakenings. However, no significant differences have been observed in any of the asthma symptoms before and after intervention between the two groups.

McPherson [15] undertook a pilot study and demonstrated increased knowledge about triggers of asthma after using the computer-assisted program known as 'The Asthma File'. This study further indicated that children are able to extract relevant information from multimedia sources when they are encouraged to participate actively in their learning. Porter et al [16] reported that deployment of a parent-driven decision support system may improve quality of asthma care and patient satisfaction. Krishna et al [17] conducted a randomized controlled trial and reported a significant increase in asthma knowledge of children and their caregivers; decrease in asthma symptom days (81 days vs. 51 days per year); and decrease in number of emergency department visits (1.93 vs. 0.62 per year) through the use of an internet-based multimedia asthma education program. Krishna et al [17] also showed that the intervention group used a significantly lower average daily dose of inhaled corticosteroids (434 vs. 754 micro gram). Homer et al [18] demonstrated that computerized education programs substantially reduced emergency department and office visits as well as improved asthma-related knowledge.

### Cognitive Disability (7 studies)

Seven studies assessed the impact of applications on mental disabilities such as ADHD (attention deficit hyperactivity disorder); dyslexia; learning and behavior problems; and learning disabilities. Interventions considered in the studies were computerized training of working memory [19], visual hemisphere-specific stimulation [20], and software for computer-delivered instruction [21], computer-based reading [22], computerized study guide [23], computer-assisted mathematics computation drill-down-practice [24], and computer-assisted instructional software [25]. These studies also focused on outcome measures such as neuropsychological assessments [19], substantive errors, fragmentations, and reading time [20], compliance, visual discrimination task, and collateral behavior [21], spelling skill, and reading skill [22], multiple choice reading passage tests, note taking [23], computation skills in addition, subtraction, multiplication, and division [24], and multiple choice tests [25]. Improvements and significant treatment effects were reported in relation to reduction in symptoms of inattention and hyperactivity/impulsivity and improvement for the span-board task, working memory deficits, response inhibition, and complex reasoning [25]. Other reported improvements included fewer substantive errors and more fragmentations on a text-reading task compare to control group [20], improved accuracy and fluency during reading and spelling [22], improvement in math computation performance over time [24], higher quiz scores and higher overall test scores for a computer study guide group compared to a lecture group [25].

### Autism (9 studies)

Results from a study by Bernard-Opitz et al [26] demonstrated significant improvement in vocal imitations when participants used computer-assisted instruction instead of traditional play interactions. Williams et al [27] found that computer-instructed learning is more effective than book based learning, and children with autism spend more time on reading material when they access it through computer compared to equivalent materials in book format. Findings from another study by Bernard-Opitz et al [28] suggested that computer-assisted instruction is effective in teaching problem-solving skills to children with autism. It also showed that normal and autistic preschool children can be taught social problem solving skills using animated solutions presented by a computer. Swettenham [29] reported that computerized instruction programs are effective in enhancing cognitive functions. Moore et al [30] suggest that participants who received computer-assisted instruction program were more attentive, motivated and learned more vocabulary words than those who received teacher-presented behavioral treatment.

Stenens et al [31] showed that six weeks of high-intensity training with a computerized intervention program (originally designed to improve language skills) influenced neural mechanisms of selective auditory attention in children with language impairment. In another study, these authors showed that computer-based problem solving instructional programs significantly improved math and problem solving ability of students with learning disabilities [32]. Kast et al [33] showed that a three-month computer-based training significantly improved the writing skills of children with and without developmental dyslexia. A significant impact of a computerized progressive attention training program on improving reading comprehension and passage copying of children with ADHD is shown by Shalev et al [34].

### Pediatric oncology (3 Studies)

In a before-and-after study conducted in an academic medical centre from 2001 to 2004, Kim et al [35] showed a significant reduction in ordering errors in pediatric chemotherapy through the use of a computerized provider order entry system. Other studies showed a reduction in prescription errors of chemotherapy dosage using a web-based decision-support system (Leukemia Intervention Scheduling and Advice, 'LISA") [36]; and improved patient education with a computer-assisted instructional program [37].

### Diabetes (2 studies)

Zahlmann et al [38] found a significant decrease in HbA_1c _values with a decision support system ('DIABETEX') and Horan et al [39] reported a significant improvement in the level of blood glucose with the use of a computer-assisted system designed to promote self-management of insulin.

### Other Studies (3)

Other researchers looked at patients with bronchiolitis, headache or juvenile idiopathic, and brain injury. The studies described the use of interventions such as clinical evidence modules integrated into computerized provider order entry [40]; electronic diaries [41] and web-based interactive interventions [42].

Other studies showed benefits associated with a computerized provider order entry on the frequency of ordering antibiotics, bronchodilators and corticosteroids [40]; increase in the return rates and accuracy of records using electronic diaries [41], and improvement in child anti-social behavior problems and reduction in conflict with parents in relation to school issues using a web-based intervention [42].

## Discussion

Our findings suggest that information and communication technologies can indeed improve quality of care and have an overall positive effect on adherence to clinical guidelines; patient knowledge and self-efficacy; decrease rate of emergency room and medical office visits; and improved patient safety through a reduction in errors. However, little is known about the effects of information technology on resource utilization.

As reported by a previous systematic review, there are encouraging results in relation to pediatric asthma [9]. Studies on autism and learning disabilities demonstrated positive effects of computer-assisted programs in behavioral and cognitive outcomes. Our review also suggests that children with chronic diseases from pre-school through high-school age can respond positively to computer-based education and disease management programs. For children, guardians and health care providers who participated in the reviewed studies, computer-based interventions were effective in improving health care behavior and health care outcomes, in enhancing knowledge and communication with parents and health care providers, and in reducing the need for urgent medical care. This is consistent with findings of systematic reviews of studies including adults with chronic conditions [2,43,44].

Computer-based intervention studies have also been reported to be effective in preventing obesity. Frenn et al [45] examined the effectiveness of internet/video delivered intervention to increase physical activity and reduce dietary fat among low-income, seventh-grade students with various cultural backgrounds. The findings of this study further add to the body of literature suggesting that computer-based interventions are effective in improving health behaviors in middle school students.

The studies available seem to support, consistently, that patient education and disease management can be facilitated through computer-assisted programs and health care related websites. Moreover, these comprehensive interventions are effective in engaging patients and in improving their health and well-being. Others have also demonstrated that when individuals with chronic disease receive effective and coordinated care on an ongoing basis, the need for costly emergency room visits and hospital admissions reduce significantly.

The results of this systematic review suffer from some limitations. For example, a key issue that cannot be addressed with the available data is whether or not the knowledge uptake from the various applications improved soon after initiation of the intervention, and also whether or not there is long-term impact. Longitudinal studies over extended periods of time are required to answer such questions. Also some of the outcome variables such as reduced ED visit and lower dose of medications will need to be adjusted for potential confounders (e.g., disease severity, concomitant medications, and so on). This information is generally not reported.

Reviews of the medical informatics literature pertaining to the treatment of adults with chronic conditions suggest that human factors are at least as important as the technological elements powering the intervention itself. For our study, the majority of users of health informatics interventions were school age children who either directly used computers or the Internet, or who were directly exposed to interventions in several short sessions at school. Issues related to human factors were not discussed in any detail in the papers reviewed.

## Conclusion

In conclusion, our systematic review has revealed several important applications of biomedical informatics in pediatric chronic diseases. Published studies suggested positive impacts of informatics predominantly in pediatric asthma. Our review has also highlighted important future directions for research. It is clear, for instance, that rigorous studies are needed to gain a better understanding of the role of informatics in inpatient settings, as well as their impact on workflows, organizational structures, health professional and patient roles, costs and equitable access to health services among members of disadvantaged communities.

## Competing interests

The authors declare that they have no competing interests.

## Authors' contributions

FM contributed to the design of the study, acquisition, analysis and interpretation of data and drafted the manuscript. RM contributed to the design of the study, acquisition, analysis and interpretation of data and helped draft the manuscript. ARJ, JSH and TT helped with interpretation of data and critical revision of the manuscript for important intellectual content. JB conceived the study and contributed to the design of the study, analysis and interpretation of data and helped draft the manuscript. All authors have read and approved the final manuscript.

## Pre-publication history

The pre-publication history for this paper can be accessed here:

http://www.biomedcentral.com/1472-6947/9/22/prepub

## Supplementary Material

Additional file 1 **Table 1**. Characteristics of included studies. Click here for file
