# Supplementary material for: Application of biomedical informatics to chronic pediatric diseases: a systematic review
Source: BMC Med Inform Decis Mak. 2009 May 5;9:22. doi: 10.1186/1472-6947-9-22 (PMC2681448; doi:10.1186/1472-6947-9-22)
Supplement: Additional file 1 — Table 1. Characteristics of included studies. [file 1472-6947-9-22-S1.doc]

Additional file 1:

Table 1: Characteristics of included studies

| Author  (Year) | Study  design | Age range/  mean  (Yrs) | Sample Size | % Male | Chronic  condition | Settings | Domain | Intervention | Comparator | Primary outcomes | Study effect | Primary Users |
| --- | --- | --- | --- | --- | --- | --- | --- | --- | --- | --- | --- | --- |
| Homer  2000 | Randomized Control Trial  (RCT)  Prospective | 3-12 | 137 | 69% | Asthma | Outpatient & Emergency department | Patient education | Interactive Computer-based  Educational program | Non-computerized educational program | Rate of ER & outpatient visits | + | Patients |
| McPherson  2002 | Crossover  Prospective | 7-14 | 10 | 100% | Asthma | Outpatient | Patient education | Computer program | No educational program | Knowledge of asthma triggers | + | Patients |
| Huss  2003 | RCT  Prospective | 7-12 | 110 | 44% | Asthma | Outpatient | Patient education | Computer game | Conventional education | Improvement in asthma symptoms | - | Patients |
| Porter  2006 | RCT  Pre-Post  Prospective | 1-12 | 286 | 63% | Asthma | Outpatient & Emergency Department | Disease management | Patient-Centered decision support  (asthma kiosk) | Conventional care | Quality of care and patient satisfaction | + | Parents & or Primary care givers |
| Chan  2003 | RCT  Prospective | 6-17 | 10 | 50% | Asthma | Outpatient | Disease management | interactive web- base education | In-person education | Disease management and rate of ER visits | + | Patients |
| Krishna  2003 | RCT  Prospective | 7-17 | 228 | 65% | Asthma | Outpatient | Disease management | internet asthma education | Verbal and printed education material | Asthma knowledge & Rate of ER visits | + | Patients |
| Bartholomew  2000 | RCT  Prospective | 6-17 | 133 | 65% | Asthma | Outpatient | Disease management | Multi media CD ROM application | Usual care | Asthma knowledge and rate of ER visits | + | Patients |
| Shegog  2001 | RCT  Prospective | 8-13 | 71 | 65% | Asthma | Outpatient | Disease management | CD-ROM | No Intervention | Asthma knowledge, self-efficacy &  treatment | + | Patients |
| Palermo  2004 | RCT  Prospective | 8-16 | 60 | 30% | Arthritis and headache | Outpatient | Monitoring or prevention | Electronic pain diary | Paper pain diary | Compliance | + | Patients |
| King  2007 | RCT  Before-After  Prospective | 0-2 | 334 | 56% | Bronchiolitis | Inpatient | Therapy or Guidelines | Clinical evidence module integrated in to CPOE | Conventional treatment | Frequency of ordering antibiotics, bronchodilators & corticosteroids | + | Medical residents |
| Wade  2006 | Intervention | 5-16 | 6 | 67% | Traumatic brain injury | Outpatient | Therapy | Web-based intervention | Conventional treatment | Child behavior problem, social competence | + | Families |
| Zahlmann  1990 | RCT  Pre-Post  Prospective | 5-16 | 21 | 62% | Type I diabetic | Outpatient | Therapy or Guidelines | Decision support system (Diabetex) | Conventional  treatment | HbA_1c_ | + | Clinicians |
| Horan  1990 | RCT  Match case  Prospective | 12-19 | 20 | 30% | Diabetes | Outpatient | Disease management | Computer-based Diabetes Self-Control System (DISC) | Conventional education ( printed material) | Blood glucose | + | Patients |
| Plienis  1985 | RCT  Before-After  Prospective | 4-14 | 17 | - | learning and behavior problems | Outpatient | Therapy | computer-delivered instruction | Adult- delivered instruction | Total Disruptive Score | + | Patients |
| Moore  2000 | RCT  Prospective | 3-6 | 14 | 86% | Autism | Outpatient | Therapy | Computer-assisted instruction program | Teacher presented behavioral treatment | Vocabulary acquisition | + | Patients |
| Swettenham 1996 | RCT  Prospective | 3 | 24 | - | Autism | Outpatient | Therapy | Computer-assisted instruction program | Normal children | Development of theory of mind-Understanding of false belief | + | Patients |
| Williams  2002 | RCT  Crossover  Prospective | 3-5 | 8 | - | Autism | Outpatient | Therapy | Computer-instructed  learning | Personal instruction (book learning) | Time on task | + | Patients |
| Bernard-Opitz  2001 | Matched case-control | 4-8 | 16 | 69% | Autism | Outpatient | Therapy | Computer-assisted instruction | control | Improvement in Social Problem solving | + | Patients |
| Bernard-Opitz  1999 | RCT  Prospective | 3-7 | 10 | 90% | Autism | Outpatient | Therapy | Computer-assisted instruction | Personal instruction | Vocal imitation | + | patients |
| Klingberg  2005 | RCT  Multicentre  Prospective | 7-12 | 53 | 83% | ADHD | Outpatient | Patient education | Computer program | Comparison program, easier version | Visuospatial working memory task | + | Patients |
| Van Strien  1995\ | RCT  Prospective | 8-12 | 40 | 60% | Dyslexia | Outpatient | Therapy | Visual hemisphere-specific stimulation | L-type dyslexia | Reading accuracy | + | Patients |
| Van Daal  1992 | RCT  Pre-Post  Prospective | 9.7 | 28 | 61% | Learning disability | Outpatient | Therapy | Computer –based reading and spelling program | - | Reading and spelling skills | + | Patients |
| Horton  1989 | RCT Prospective | - | 31 | - | Learning disability | Outpatient | Computerized study guide | Computerized study guide | Remedial | Neutral group | + | Patients |
| Stevens 2008 | RCT Prospective | 7.5 | 33 | 51% | Learning disability | Outpatient | Training | Computerized Intervention | Control | Standardized language assessment | + | Patients |
| Miranda-Casas 2008 | RCT Prospective | 8-10 | 44 | - | Mathematics Learning disability | Outpatient | Therapy | Computer-assisted instructional program | Control | Solving problems tests | + | Patients |
| Kast 2007 | Matched case-control | 9-11 | 80 | 60% | dyslexia | Outpatient | Therapy | Computer-based writing traning | Control | Writing skills | + | Patients |
| Shalev 2007 | RCT  prospective | 6-13 | 36 | 83% | ADHD | Outpatient | Therapy | Computerized progressive attentional training | Control | Reading comprehension | + | Patients |
